# Spontaneous-Onset Delayed Spinal Arachnoiditis With Dorsal Cord Herniation in a 29-Year-Old Paraplegic Patient: A Case Report

**DOI:** 10.7759/cureus.51374

**Published:** 2023-12-31

**Authors:** Kuldeep Bansal, Mayukh Guha, Anuj Gupta

**Affiliations:** 1 Spine Services, Indian Spinal Injuries Center, New Delhi, IND; 2 Spine Surgery, Max Superspeciality Hospital, New Delhi, IND

**Keywords:** spinal cord injury, rehabilitation, paraplegic, spinal cord herniation, spinal arachnoiditis

## Abstract

Spinal adhesive arachnoiditis is a rare occurrence with a diverse etiology. The clinical picture is not universal, and varying degrees of neurodeficit have been mentioned. Spontaneous spinal cord herniation or idiopathic spinal cord herniation occurs due to displacement of the cord through a dural or arachnoid defect.

We report a case of a 29-year-old male paraplegic patient with a nontraumatic spinal cord injury (SCI) following surgery for an intradural extramedullary lesion at T10-T11 level who developed loss of truncal balance after two years of the index surgery. After a thorough clinical examination and MRI as well as other investigations, the patient was diagnosed as having spontaneous-onset delayed spinal arachnoiditis with dorsal cord herniation through the laminectomy window with effacement of neural tissue and ascending edema up to T6 level.

A new-onset weakness or the development of an ascending loss of sensory level with a loss of truncal balance should alarm the therapist about some new pathology happening at the cord level in patients with SCI. In this regard, spinal adhesive arachnoiditis with or without cord herniation should always be suspected in a paraplegic patient with delayed-onset deterioration of neurology. Differential diagnoses like arachnoid web and arachnoid cysts should also be kept in mind.

## Introduction

Adhesive arachnoiditis of the spinal cord is a relatively rare entity but can cause a significant amount of distress to the patient. The understanding of its pathophysiology is still evolving, but the distinguishing features are the non-specific inflammation of the arachnoid, leading to adhesion and fibrosis around nerve roots and other neural elements [1]. Sequential aggregation of neural elements blocks the cerebrospinal fluid (CSF) and vascular flow, leading to clinical manifestations. The etiologies have been diverse, like idiopathic, iatrogenic, degenerative, infectious, traumatic, etc. [2, 3]. In the early literature, spinal arachnoiditis most commonly affected cervical and thoracic levels; most of them were post-infective (predominantly post-tubercular) [4]. Also, cases of contrast media used for myelography have been cited as a causative factor. Most recently, cases of lumbar arachnoiditis have been reported following epidural injections and lumbar spine surgery [4,5]. The clinical picture is not universal, and varying degrees of neurodeficit have been mentioned. An MRI with or without contrast is the gold standard imaging modality to detect arachnoiditis, but it lacks generalized, accepted consensus regarding the distinguishing features [6].

Spontaneous spinal cord herniation or idiopathic spinal cord herniation occurs due to displacement of the cord through a dural or arachnoid defect. Post-surgery cord herniation is a known occurrence, usually seen dorsally in the cervical spine after laminectomy. In the thoracic spine, the cord herniation is seen ventrally due to a dural defect that allows the communication of subarachnoid space with extradural space. Normal thoracic kyphosis allows very close contact of the thoracic cord with the ventral dura, allowing cord herniation ventrally [7].

We report a case of spontaneous-onset delayed spinal adhesive arachnoiditis with dorsal cord herniation through the laminectomy window in a 29-year-old male paraplegic patient in whom arachnoiditis developed two years after thoracic spine surgery.

## Case presentation

**Figure 1:**
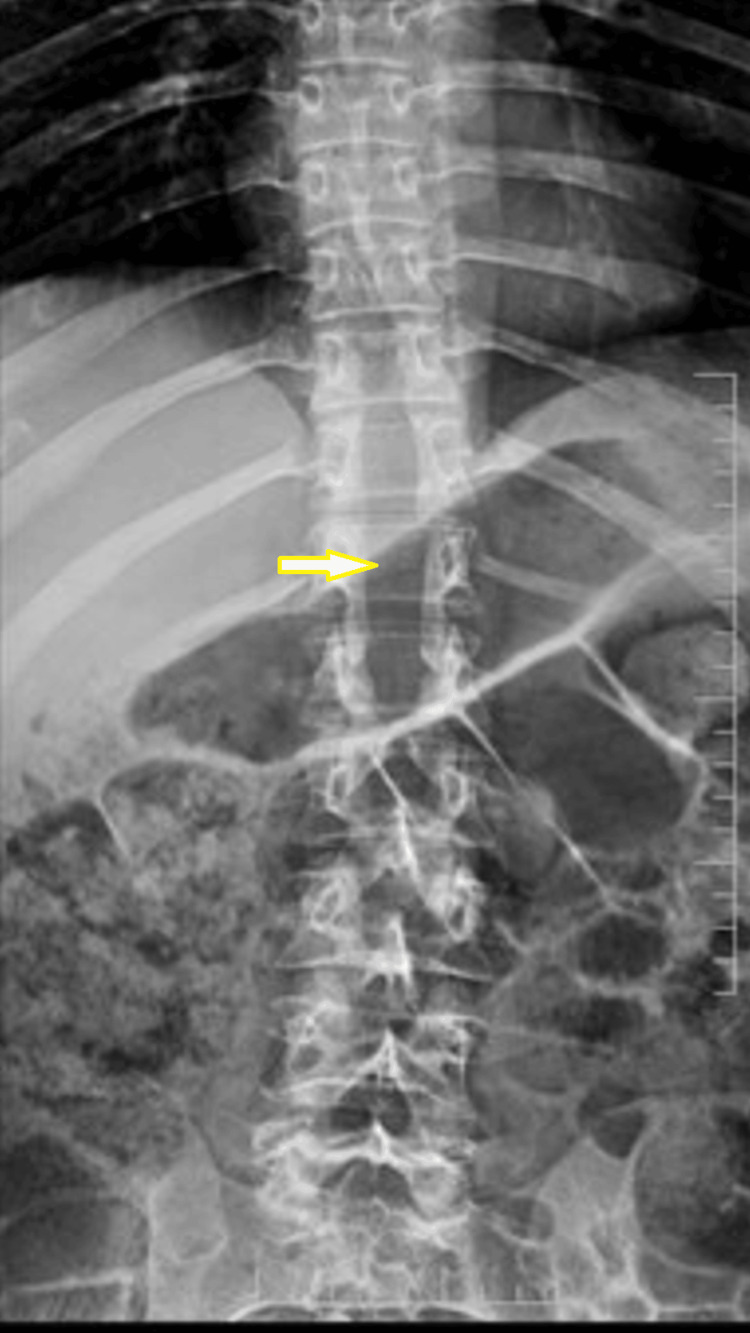
X-ray of the thoracolumbar spine (anteroposterior view) showing the laminectomy window in T9-T12 (yellow arrow) from the previous surgery

A 29-year-old male patient was operated on elsewhere for an intradural extramedullary lesion two years ago. Midline laminectomy from T9 to T12 was done as shown in the radiographs (Figure 1), and the lesion was removed.

The patient's neurological status assessment was done according to the American Spinal Injury Association (ASIA) and the International Standards for Neurological Classification of Spinal Cord Injury (ISNCSCI) grading. The sensory level was T8, the motor level was T8, and the neurological level was T8 with the ASIA Impairment Scale (AIS) grade A. Anti-tubercular therapy was started after the surgery for nine months. Unfortunately, there was no improvement in his neurology; he remained paraplegic and visited our center for rehabilitation. Goal planning was done, and structured rehabilitation was started in the form of sitting, balance training, and wheelchair-assisted mobilization. Bladder and bowel training were also commenced.

**Figure 2:**
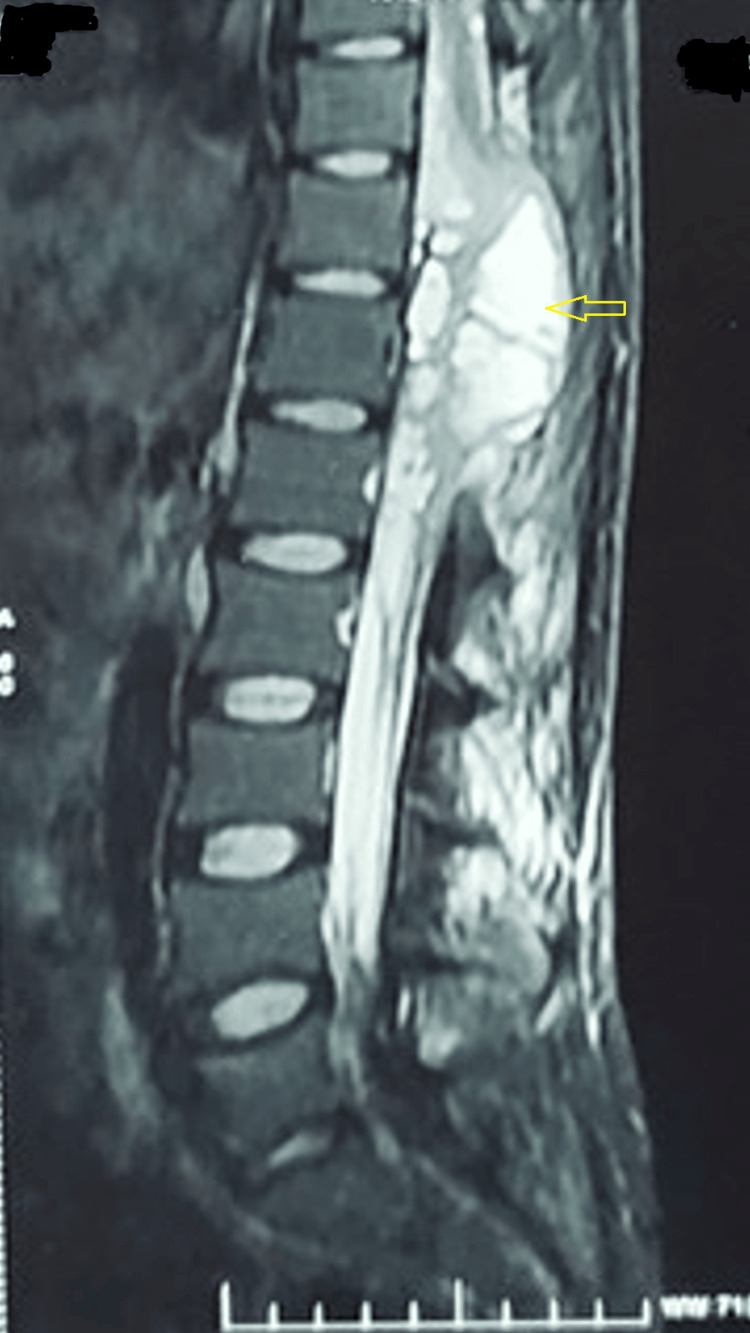
An MRI of the thoracolumbar spine (mid-sagittal image) Loculated fluid collection with contrast enhancement in the spinal canal extending from T9 to T12 (yellow arrow) vertebral levels involving the distal cord, conus medullaris, and cauda equina nerve roots

**Figure 3:**
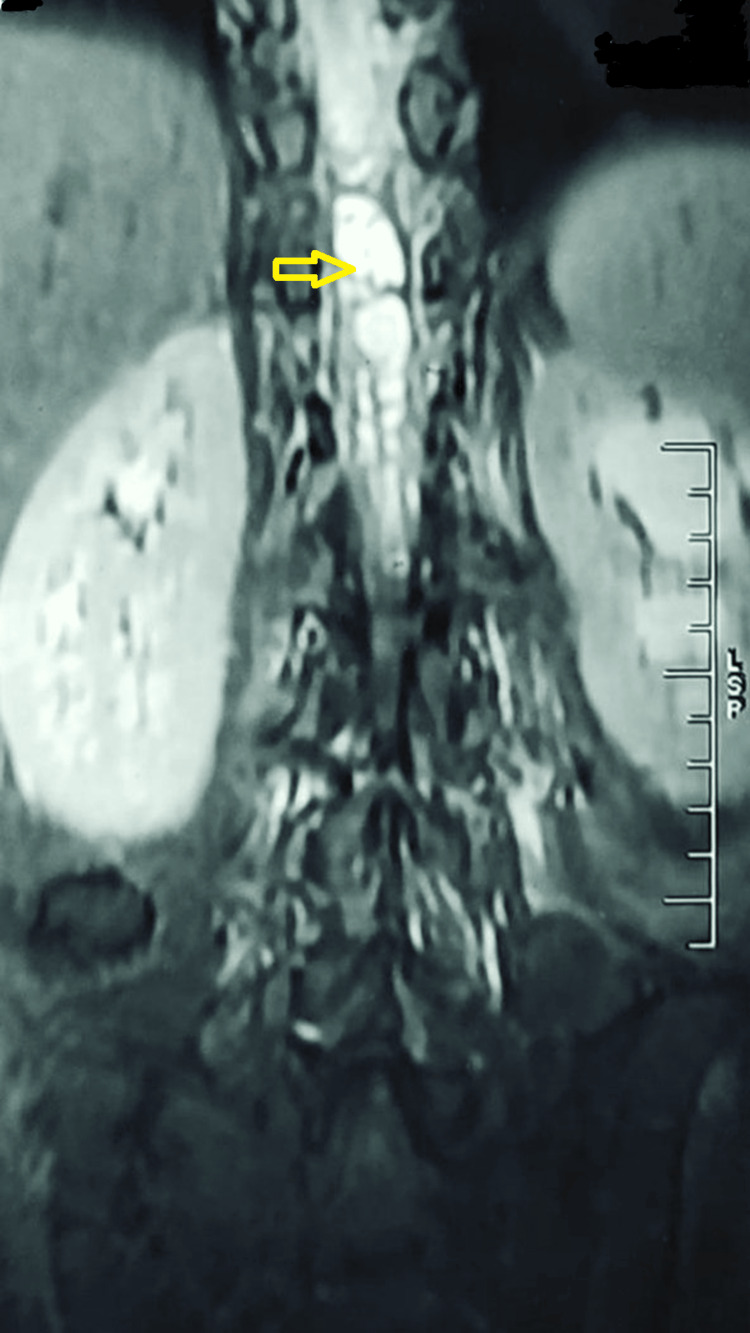
An MRI of the thoracolumbar spine (mid-coronal image) Loculated fluid collection with contrast enhancement in the spinal canal extending from T9 to T12 vertebral levels (yellow arrow) involving the distal cord, conus medullaris, and cauda equina nerve roots

During the hospital stay, the patient experienced an ascension in his sensory level, which was T8 at the time of admission. It ascended to T6 and then to T4, with an associated truncal imbalance, making him unable to proceed with balance training. A detailed neurological assessment was done, which revealed the sensory level to be T4. An MRI of the thoracic spine with whole spine screening was done, and it showed 2.5 x 2.7 x 7.3 cm of loculated fluid collection around the spinal canal extending from T9 to T12 vertebral levels involving the distal cord, conus medullaris, and cauda equina nerve roots (Figures 2-3).

**Figure 4:**
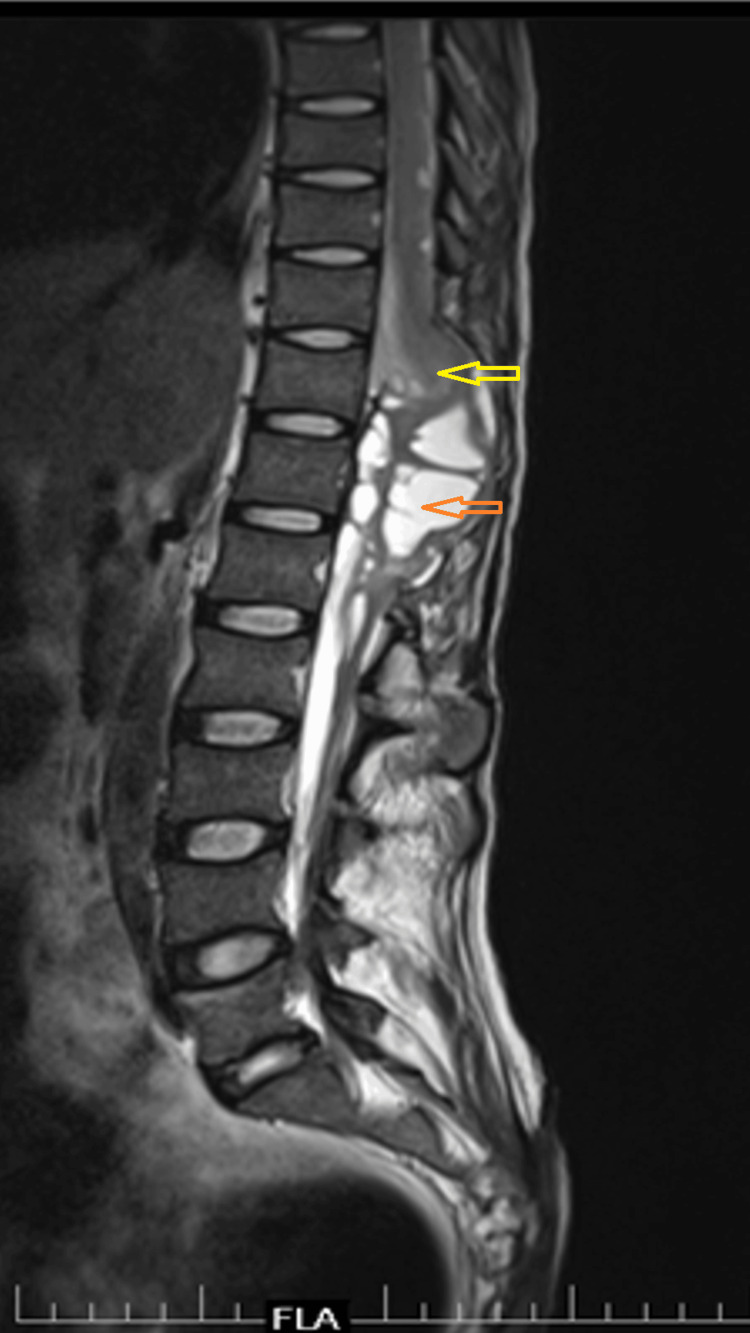
An MRI of the thoracolumbar spine (mid-sagittal section) Features of arachnoiditis, loculated cerebrospinal fluid (CSF) collection (orange arrow), and dorsal spinal cord herniation (yellow arrow) with effacement of all the neural tissue are seen.

**Figure 5:**
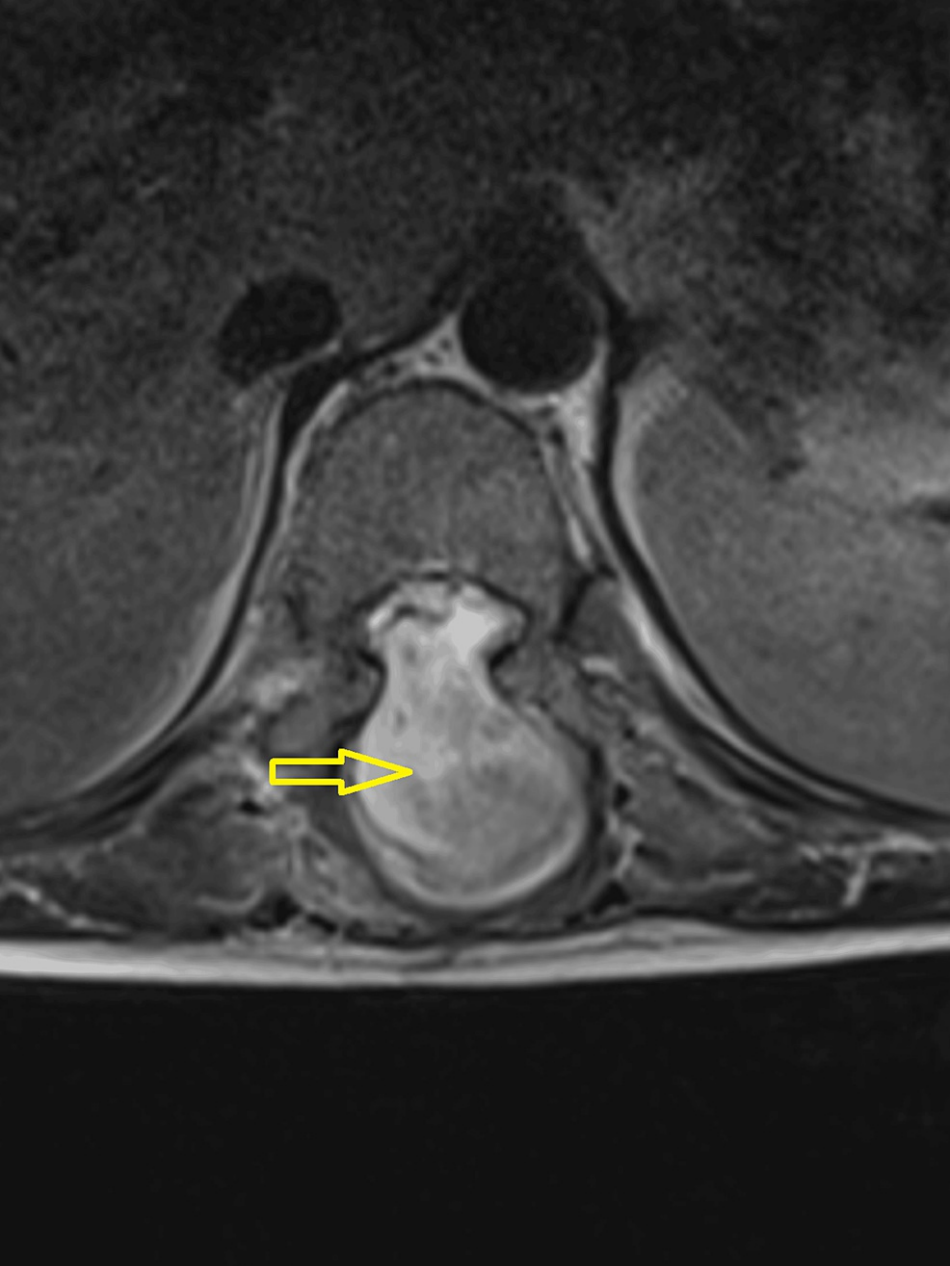
An MRI of the thoracolumbar spine (axial images) Features of arachnoiditis, loculated cerebrospinal fluid (CSF) collection, and dorsal spinal cord herniation with effacement of all the neural tissue (yellow arrow) are seen.

**Figure 6:**
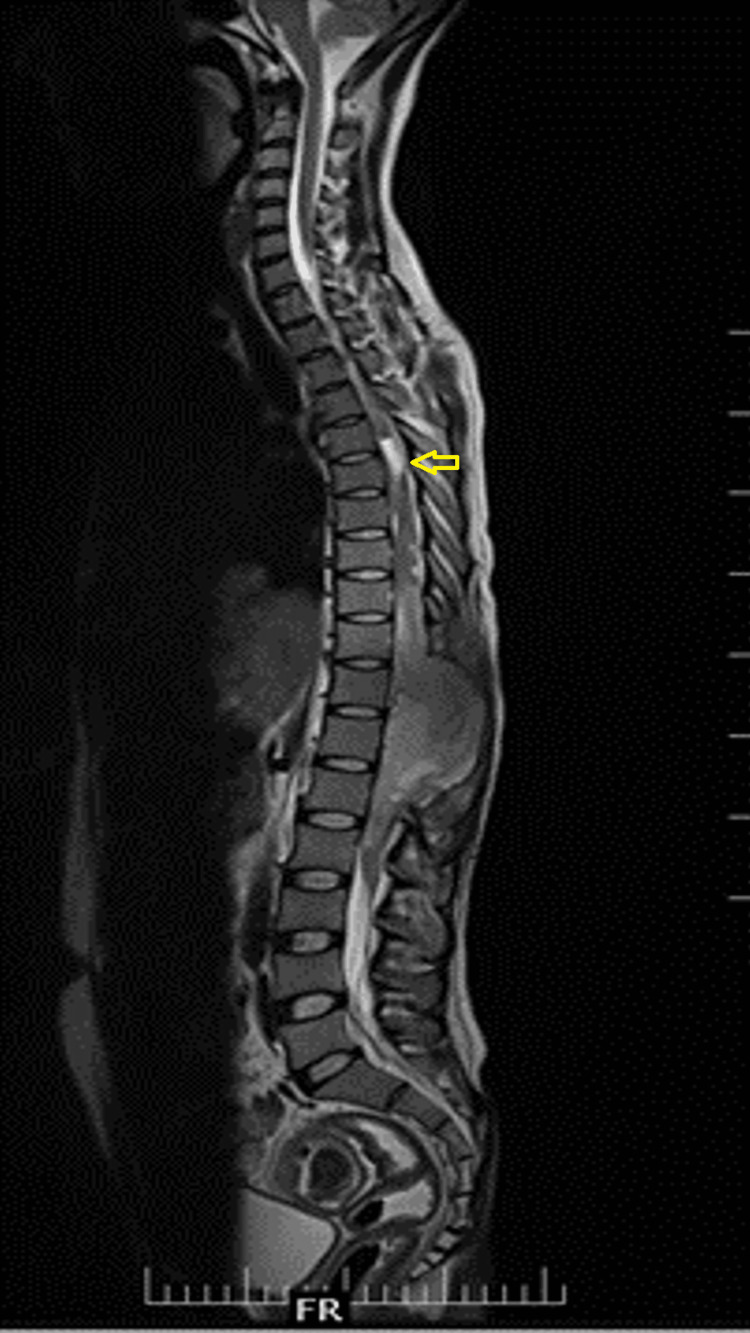
An MRI of the whole spine screening (T2-weighted mid-sagittal image) shows cord edema ascending to the T6 level (yellow arrow).

There was an extension of the collection into the posterior paraspinal soft tissue through the laminectomy window. Cord edema was also appreciated up to the T6 level (Figures 4-6).

**Figure 7:**
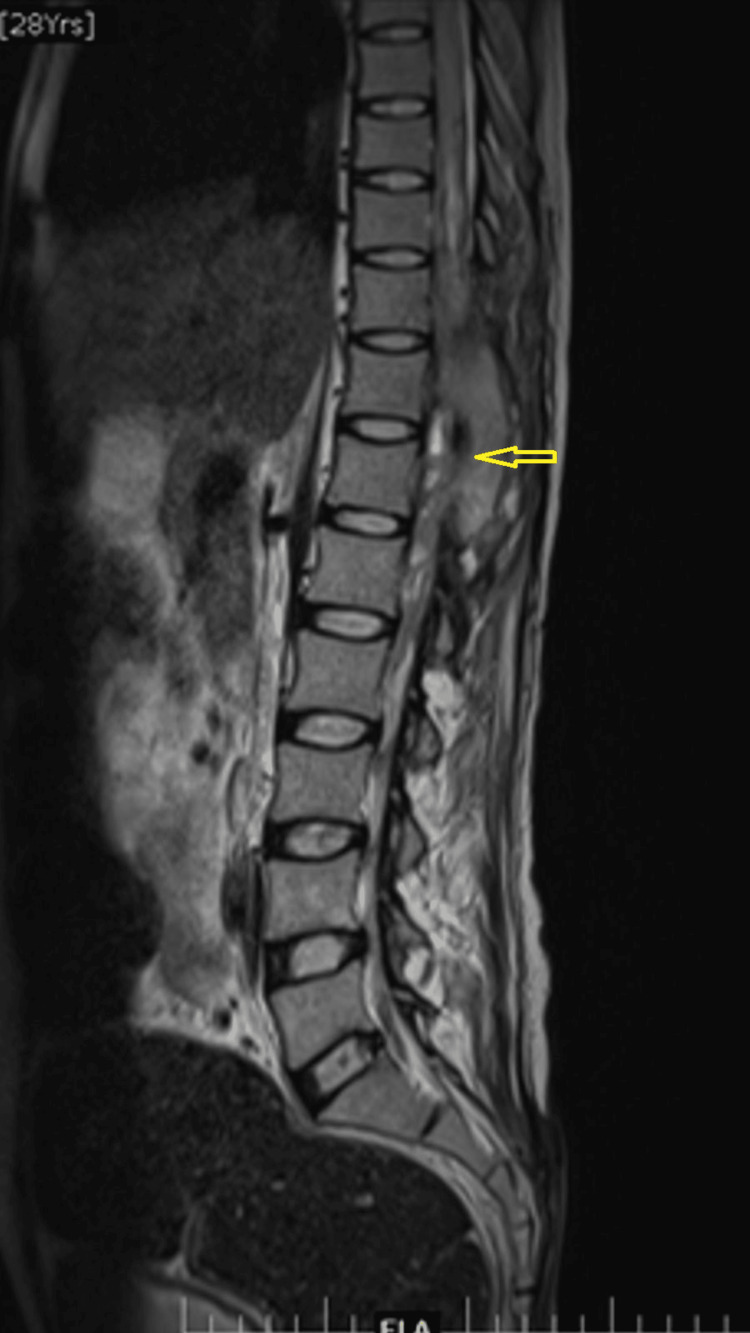
An MRI of the thoracolumbar spine after six weeks of treatment showed a decrease in cord edema, resolution in collection, and herniation of the cord (yellow arrow).

Features were suggestive of arachnoiditis with dorsal cord herniation through the laminectomy window with a loculated cyst. The blood picture showed neutrophilic leucocytosis, elevated erythrocyte sedimentation rate (ESR), and C-reactive protein. Aspiration of CSF was done, and culture showed a methicillin-resistant *Staphylococcus aureus* (MRSA) infection. The patient was started on an injection of vancomycin according to drug sensitivity, along with intravenous steroids (methylprednisolone). On serial CSF and blood investigations, biochemical markers gradually improved. The patient also showed clinical improvement; his sensory level receded to T8, and he regained his truncal strength and balance. Repeat MRIs at three weeks and six weeks showed a decrease in cord edema, resolution in collection, and herniation of the cord (Figure 7).

## Discussion

Spinal adhesive arachnoiditis and its sequelae are not very common in the literature. Spinal arachnoiditis with dorsal cord herniation through the laminectomy window is also not common in paraplegics with AIS-A neurology and has not been reported to the best of our knowledge. The basic feature is the inflammation of the meningeal linings, which ultimately spreads to both the arachnoid and dura, nerve roots, and spinal cord. At the cellular level, fibrosis, adhesion, and blockade of blood flow and CSF circulation are the ultimate results [2, 4]. It's a difficult entity to diagnose even in patients with intact neurology; expecting it in a young paraplegic patient with no inciting factor is very difficult. An MRI has become the investigation mode of choice, but there has often been controversy regarding the diagnostic MRI picture [1]. The globally accepted features nowadays are the centrally or peripherally located adherent mass of nerve roots in the thecal sac, enhancing mass or mass-like collection replacing the subarachnoid space, or empty thecal sac with nerve root adhesion to the dural lining [6]. Added pathologies like cord herniation and CSF collection make diagnosing even more challenging.

Since the era of post-tubercular arachnoiditis in the late 1970s and 1980s, the focus has been shifted to epidural injections, caudal blocks, and local anesthetic procedures as the causative factors of arachnoiditis. Some studies have also mentioned delayed postoperative arachnoiditis, but it is very rare [8]. One study reported adhesive arachnoiditis two and a half months after the surgical stabilization of a thoracolumbar fracture [9].

In our case, there was a delayed onset (two years after the spine surgery) of spontaneous spinal arachnoiditis of the mid-lower thoracic level and subsequent dorsal migration of the spinal cord along the laminectomy window with a loculated CSF collection and cord edema, leading to truncal imbalance in a 29-year-old paraplegic patient. Spontaneous spinal arachnoiditis, too, after this much delay, is very rare and has been reported for the first time, according to the best of our knowledge. Along with that, cord herniation at the thoracic level is most commonly seen ventrally or ventrolaterally, not dorsally, due to normal thoracic kyphosis [10]. Le et al. presented a case of a 55-year-old man presenting with progressive low back pain and a sensorimotor deficit of the left lower limb, with imaging revealing a dorsal dural defect with tethering and herniation of the spinal cord at T7 [11]. This was the first time a dorsal cord herniation in the thoracic spine was reported. In that regard, our case also demands special attention, as it is the second case that documents the same.

Ascending neurological deterioration in a spinal cord injury (SCI) patient (traumatic or nontraumatic) is difficult to diagnose. When it happens to a paraplegic patient, it becomes even more tricky to find out. While acute deterioration (within the first hours or days) is the most common, subacute or late deterioration is relatively rare [12]. Late deterioration (at least two months after injury) is attributed to syrinx formation, post-traumatic myelomalacia, late cord edema, etc. [12, 13]. We are reporting late-onset spontaneous spinal arachnoiditis two years after the initial surgery with dorsal herniation of the thoracic cord and a loculated CSF collection presenting with an ascending sensory and motor deficit. The symptoms were very complex, and it was even more difficult to diagnose in a paraplegic patient. Conservative management was done in the form of intravenous steroids and antibiotics, and the patient responded very well.

This late-onset deterioration of neurology in a paraplegic patient was due to spinal adhesive arachnoiditis and the resultant alteration of CSF circulation, leading to loculated CSF collection. This alteration of CSF circulation has been attributed as a cause of delayed ascending neurodeficit in the literature quite extensively [14, 15]. In paraplegic patients, diagnosing an ascending deficit can only be done by assessing the sensory level and identifying a loss of truncal rotation and balance. Physiotherapists and occupational therapists should be watchful regarding sensory levels and must be trained in this fashion.

There are two main differential diagnoses of MRI pictures of adhesive arachnoiditis with displacement or herniation of the spinal cord: arachnoid cyst and dorsal arachnoid web. Secondary arachnoid cysts are commonly due to trauma, surgery, or iatrogenic injections or procedures in and around the subarachnoid space. An MRI shows CSF intensity without contrast enhancement, and phase contrast imaging depicts decreased CSF flow within the cyst [16]. An arachnoid web is a thickened band of arachnoid over the dorsal aspect of the cord. An MRI shows focal dorsal indentation of the cord by the band and subsequent anterior displacement of the thoracic cord, leading to the widening of the CSF space dorsally, known as the scalpel sign [17]. A variety of neurological symptoms, from gait instability to episodic weakness with an ascending sensory level, have been reported for the first time, as seen in our case. In our case, there was a loculated CSF collection with dorsal cord herniation, which subsided after conservative management with intravenous antibiotics and steroids.

Proper management guidelines for adhesive arachnoiditis and cord herniation are still not established. Surgeries have been done depending on the symptoms and causative pathology. Procedures like expansive duraplasty, microscopic adhesiolysis, myelotomy, intraventricular drain placement, and spondylodesis have been tried [18, 19]. However, the long-term outcome of surgical treatment in these cases is not satisfactory. Conservative management with corticosteroids has proven to be useful in the early stages of spinal arachnoiditis but not in the later stages [19]. In our case, we commenced intravenous steroids very early in the disease along with proper antibiotics, which helped in the timely resolution of symptoms.

## Conclusions

Spinal adhesive arachnoiditis should always be suspected if there is a delayed worsening of neurodeficits or a new-onset neurological deficit, even in paraplegics. Rehabilitation plays a pivotal role in paraplegics to make them independent and for community integration. Normally, autonomic dysreflexia, bowel and bladder infections, chest infections, and pressure sores create hurdles for the patient with a spinal cord injury. However, ascending sensory levels and loss of truncal balance might be early symptoms of arachnoiditis in paraplegics, which should be given due consideration, and therapists should be aware of these conditions. Differential diagnoses of spinal cord herniation, like arachnoid web or arachnoid cyst, should always be kept in mind before starting treatment. Conservative treatment in the form of steroids may be beneficial in the early stages of the disease. A variety of surgical options are also available, but none have proven superior to others. Spontaneous-onset delayed spinal arachnoiditis with dorsal cord herniation through laminectomy window deficit at the thoracic level is very rare and reported for the second time, according to our knowledge.
